# Comparative Analysis of Lateral Preferences in Patients With Resistant Schizophrenia

**DOI:** 10.3389/fpsyt.2022.868285

**Published:** 2022-04-05

**Authors:** Georgi Panov

**Affiliations:** Psychiatric Clinic, University Hospital for Active Treatment “Prof. Dr. Stoian Kirkovic”, Trakia University, Stara Zagora, Bulgaria

**Keywords:** schizophrenia, resistance, cross-domination, left-handed, right-handed, mixed lateralization, eye dominance, functional lateralization

## Abstract

**Background:**

Schizophrenia is a chronic brain disorder of diverse etiology and clinical presentation. Despite the expansion of treatment methods, between 30 and 50% of cases remain resistant to treatment. In patients with schizophrenia, specifics in the dominant lateralization in the brain function have been discovered. This gave a reason to seek the relation between functional lateralization and the effect of treatment.

**Methods:**

Of the 105 people observed with schizophrenia, 45 (42.9%) were treatment resistant, and 60 (57.1%) were considered responders. We compared functional lateralization (hand, foot, and eye) between the two groups. Handedness was ascertained by using the Edinburgh Handedness Inventory. The assessment was made at 12 weeks of treatment.

**Results:**

Of all patients with schizophrenia, 41.89% have mixed lateralization, 53.34% are right winged, and 4.76% of the patients are left winged. Resistance of the symptoms shows that 26 (57.78%) are cross-dominated, 18 (40%) are right winged, and 1 (2.22%) is left winged. In patients with clinical remission, 18 (30%) are of mixed dominance, 38 (63.33%) are right winged, and 4 (6.66%) are left winged. From the results for the separate lateralization of the hand, foot, and eye, we found a significant difference only in terms of the dominance of the eye. In 44 (41.9%) of the patients, we found dominance of the left eye. In patients with resistance, the percentage established by us is higher—at 26 (57.8%). These results indicate that the increased percentage of mixed dominance in patients with schizophrenia is mainly due to left-sided lateralization of the eye, especially in those with resistance to treatment.

**Conclusion:**

We find an increased number of patients with cross-dominance left eye dominance in patients with schizophrenia. Cross-dominance and left eye dominance are associated with a higher probability of symptom resistance than other forms of lateralization (left-handed or right-handed). The high percentage of cross-dominance is due to the high percentage of left-sided dominance of the eye.

## Background

Schizophrenia is a chronic mental illness with a heterogeneous etiology and a polymorphic clinical picture. The symptoms of schizophrenia are classified into positive, negative, and cognitive. In addition to these main symptoms, frequently anxious and affective symptoms have been identified (1). Studies that assess functional connectivity in patients with schizophrenia have shown disturbances in information transfer between brain centers, particularly between the frontal lobe and cuneus and other neuronal regions (2–4).

The grouping of the clinical manifestations is a reflection of the changes in the neuronal organization as well as the changes in the different levels of loss of connectivity (5). Studies show that the left and right hemispheres are asymmetric in their morphological structure and in the cognitive functions they mediate (6). This asymmetry seems to underlie the development of basic cognitive processes, such as language acquisition (7), and it is suggested that variations in the development of this asymmetry also contribute to the pathogenesis of schizophrenia (7, 8). T. J. Crow suggests that the genetic variation associated with the evolution of *homo sapiens* and the hemispheric dominance of language development is also associated with an increased likelihood of developing psychotic symptoms (7). Other authors also suggest that the loss of left hemispheric dominance is a central neurological feature of this disease (3, 4, 9–11).

One method of assessing lateralization is to establish the dominant hand of the subject. Hand dominance has also been shown to correlate with cerebral lateralization of language expression (12), visual processing (13), declarative memory (14, 15), and emotional processing (16). An analysis of more than 40 studies has concluded that the left hand is more common in mental illness (17). When comparing the prevalence of left-handedness among 107 patients with psychotic disorders and in patients with affective disorders and anxiety, a significant difference was observed (18). In patients with psychotic disorders, some authors find a high percentage (including schizoaffective and schizophrenia) of up to 40% left-handedness (18). Other studies have found almost two times less left handers in patients with schizophrenia (between 15 and 20%) (17). There are also reverse studies. An assessment of several hundred patients showed no difference in lateralization in patients with schizophrenia, their relatives, and the control group (19).

Schizophrenia is a disease of various etiologies, and this could be the reason for the observed deviation in dominance in different studies due to the fact that different clinical subgroups have been studied. Several analyses point to such possible differences. Left-handed and those with cross-dominance with psychotic disorders report more severe impairment in social functioning and have poorer results in psychological studies (20, 21). These results may suggest that dominance may be considered a marker for a particular subtype of psychosis.

Although dominance is a constant feature (there is continuity over time) (22), a definite approach to dominance is not established in current research practice in psychiatry. A three-modal classification is most commonly used, in which subjects are classified into categories with left, right, and mixed dominance, although there are several authors who continue to view dominance as a simple dichotomy (23, 24). The mixed group includes individuals without a clear dominance or consistent preference of the hands, i.e., no pronounced lateralization. Mixed dominance is thought to reflect left hemispheric dysfunction, which may be the result of early (prenatal or perinatal) brain damage (25, 26) or other causes of neurodevelopment (27–29). Mixed dominance, which is more common in patients with schizophrenia than in normal subjects, is even considered characteristic of schizophrenia (28). An analysis of 93 patients with schizophrenia and 105 controls revealed cross-dominance in 20% of patients with psychosis compared with 3.8% in the control group (29). Another study with almost the same number of patients with schizophrenia (30) compared with controls found that the number of patients with pure left dominance was not different from that in the rest of the control group. The authors registered an increased frequency of those with mixed dominance in patients with psychosis (three times more often than the control group). They find that mixed dominance is more pronounced in chronic cases. The results of another research study suggest that some researchers consider schizophrenia as a disorder of lateralization of cerebral function. They analyze the eye/hand relationship for dominance. A meta-analysis found that 35% of right-handers and 57% of left-handers had a dominant left eye (31). Another study assessed the dominant eye in 68 patients with schizophrenia and 118 controls. The patients were aged 17–60 years, with a predominance of males −60 people, and females, 28 people, respectively. The authors found an increased incidence of cross-dominance in patients with schizophrenia. Interestingly, male patients with schizophrenia have an increased incidence of left eye dominance, and such a relationship is not found in females, given the fact that they are the smaller group in the study (32).

An increased incidence of cross-dominance has also been reported in patients with the first psychotic episode, but also associated with more pronounced mild neurological symptoms. The mixed dominance reported by them was associated with poorer school performance as well as poorer social adaptation in the premorbid period (20).

The definition of a mixed dominance category depends on the choice of a questionnaire that divides the continuum of the coefficients for considering dominance as mixed or as more pronounced on the left or right. With a few exceptions (33, 34), most existing hand questionnaires do not have valid criteria for separating dominance. In contrast, measures based on another widely used instrument in the Edinburgh Classification (EHI) (35) have repeatedly been the subject of arbitrary division of classes according to the degree of dominance.

The analysis gives us reason to draw the following conclusions: There are many studies with conflicting results on the topic. We did not find a study to analyze the differences in lateralization in patients with resistance to treatment and in patients in remission.

Working hypothesis: Based on the different results of the studies conducted in patients with schizophrenia, it can be assumed that they were conducted in different subgroups of patients. We hypothesize that we will find differences in functional lateralization in patients with resistance to treatment and in those in remission.

## Materials and Methods

Patients (105) with schizophrenia and consecutive psychotic episode were observed in a psychiatric clinic in a hospital setting. Of these, 45 have resistant schizophrenia, and the remaining 60 are in clinical remission.

The gender breakdown showed that 66 were women and 39 were men.

Inclusion criteria for patients with resistant schizophrenia are those who have met the resistance criteria of the published consensus on resistant schizophrenia (36). They are as follows:

1. Assessment of symptoms with the PANSS and BPRS scale (37, 38).
2. Prospective monitoring for a period of at least 12 weeks.
3. Administration of at least two antipsychotic medication trials at a dose corresponding to or greater than 600 mg of chlorpromazine equivalents.
4. Reduction of symptoms when assessed with the PANSS and BPRS scale by less than 20% for the observed period of time.
5. The assessment of social dysfunction using the SOFAS scale is below 60.

The exclusion criteria are as follows:

1. Intellectual disability.
2. Organic brain damage.
3. Progressive neurological or severe somatic diseases.
4. Advanced personality change.
5. Score of MMSI below 25 points.

### Measurement of Lateralization

Lateralization of brain functions was assessed for dominance of the hand, foot, and eye.

Given the specifics of the present study, the comparative analysis of patients with chronic schizophrenia (especially those with persistent and resistant psychotic production) revealed some difficulties in making a detailed assessment of some results.

### Handedness

Participants respond to this scale (35) by indicating whether they use their right, left, or either hand for 10 common actions.

### Footedness

Chapman foot preference inventory (39) requires participants to respond by indicating whether they use their right, left, or either foot for common actions.

### Eye Dominance

In determining the dominant eye, a method, such as looking through a hole [e.g., (32)], was used. To ensure that we have selected the correct dominant eye, we trained the participants.

### Procedure

Participants completed the Edinburgh Inventory Scale, Chapman foot preference. Eye preference is determined by three (or more) times attempting to look through a hole.

Given the fact that in some of the patients with pronounced psychotic symptoms it was difficult to assess the intermediate forms of dominance, we decided to group the patients into two groups in terms of hand use, leg, and eye.

### Statistical Methods

The statistical software package SPSS, was used for statistical data processing. Because we use categorical variables, and limit group numbers, chi-square test in non-parametric tests was chosen in comparing the groups.

## Results

The mean age of patients in the group of resistant schizophrenia was 36.98 years. The minimum age is 21 years, and the maximum is 60 years.

The mean age of patients in the group of schizophrenia in clinical remission was 37.25 years. The minimum is 23 years, and the maximum is 63 years.

We do not find a difference in the mean age of the patients in the both groups at the time of the study.

### Hand Lateralization Data Showed the Following Results

Of all the 105 patients we observed, we found that the right-handed were 98 (93.3%) and the left-handed were 7 (6.7%).

From the group of patients with resistance to treatment (45), it was found that 3 (6.7%) are left-handed, and the remaining 42 (93.7%) are right-handed.

In the group of patients with clinical remission, the same process was observed, which we consider to be a coincidence in terms of the percentage distribution: 4 (6.7%) left-handed and 56 (93.3%) right-handed.

From the distribution of patients in terms of lateralization of the dominant hand, we do not find differences between the two groups of patients (f) (Table 1).

**Table 1:** Relationship between functional lateralization of the hand and the effectiveness of therapy.

			**Effect of therapy**		**Total**
			**Resistant**	**Remission**	
Lateralization hand	Left	Count	3	4	7
		%	6.7%	6.7%	6.7%
	Right	Count	42	56	98
		%	93.3%	93.3%	93.3%
Total		Count	45	60	105
		%	100.0%	100.0%	100.0%

### Foot Lateralization Data Showed the Following Results

The assessment of the lateralization of the foot in all patients (105) showed that 93 (88.6%) are right footed and 12 (11.4%) are left footed. In general, we observed a higher percentage of left-handed dominance than left-handed dominance.

In the group of patients with resistance, it was found that 41 (91.1%) have a dominance of the right foot, and 4 (8.9%) have a dominant left foot.

In the group of patients in clinical remission, we found dominance of the right foot in 52 (86.7%) and dominance of the left foot in 8 (13.3%).

No statistically significant difference was found during the statistical processing. What impressed us, and was contrary to our expectations, is that we find a higher rate of left foot dominance in patients in clinical remission compared with those with resistance, despite the lack of statistical difference (Table 2).

**Table 2:** Relationship between functional dominance of the foot and the effectiveness of therapy.

			**Effect of therapy**		**Total**
			**Resistant**	**Remission**	
Lateralization foot	Left	Count	4	8	12
		%	8.9%	13.3%	11.4%
	Right	Count	41	52	93
		%	91.1%	86.7%	88.6%
Total		Count	45	60	105
		%	100.0%	100.0%	100.0%

### Eye Lateralization Data Showed the Following Results

The assessment of the dominance of the eye in all observed patients (105) showed the following distribution: dominant right eye was found in 61 (58.1%) and dominant left in 44 (41.9%).

In patients with resistance, we registered a dominant right eye in 19 (42.2%) and a dominant left eye in 26 (57.8%).

In patients in clinical remission, we found dominance of the right eye in in 42 (70%) and dominance of the left eye in 18 (30%).

The obtained results show that we find approximately twice as often dominance of the left eye in the resistance group compared with patients in remission.

Statistical analysis showed the presence of statistical dependence with Chi square 8.150, *p* < 0.05 (^**^) (Table 3).

**Table 3:** Relationship between functional lateralization of the eye and the effectiveness of therapy.

			**Effect of therapy**		**Total**
			**Resistant**	**Remission**	
Lateralization eye	Left	Count	26	18	44
		%	57.8%	30.0%	41.9%
	Right	Count	19	42	61
		%	42.2%	70.0%	58.1%
Total		Count	45	60	105
		%	100.0%	100.0%	100.0%

### The Data in Terms of Functional Lateralization (Arm, Leg, Eye), Considered as Pure Left, Right, and Cross Dominance Showed the Following Results

Of the 105 patients studied, 44 (41.89%) have mixed lateralization, 56 (53.34%) are right-handed, and 5 (4.76%) of the patients are left-handed.

The distribution of the dominance of the lateralization in patients with resistance to treatment shows that 26 (57.78%) are cross-dominant, 18 (40%) are right-handed (hand, foot, eye), and 1 (2.22%) is left-handed (hand, foot, eye).

We found that more than half of the patients with resistant schizophrenia have mixed (cross-dominance) and an insignificant percentage are pure left-handed.

The distribution of the dominance of the lateralization in patients in clinical remission shows that 18 (30%) of the patients have mixed dominance, 38 (63.33%) are right-handed, and 4 (6.66%) are left-handed (Table 4).

**Table 4:** Relationship between pure dominance (hand,foot,eye) and cross-dominance with treatment efficacy.

			**Lateralization**			**Total**
			**Right**	**Left**	**Mixed**	
Effect of therapy	Resistant	Count	18	1	26	45
		%	32.1%	20.0%	59.1%	42.9%
	Remission	Count	38	4	18	60
		%	67.9%	80.0%	40.9%	57.1%
Total		Count	56	5	44	105
		%	100.0%	100.0%	100.0%	100.0%

The results show that cross-dominance is more common in patients with resistant schizophrenia than in those in remission. We find twice as many patients with cross-dominance in the group of patients with resistance to treatment as those in remission.

**Figure 1:**
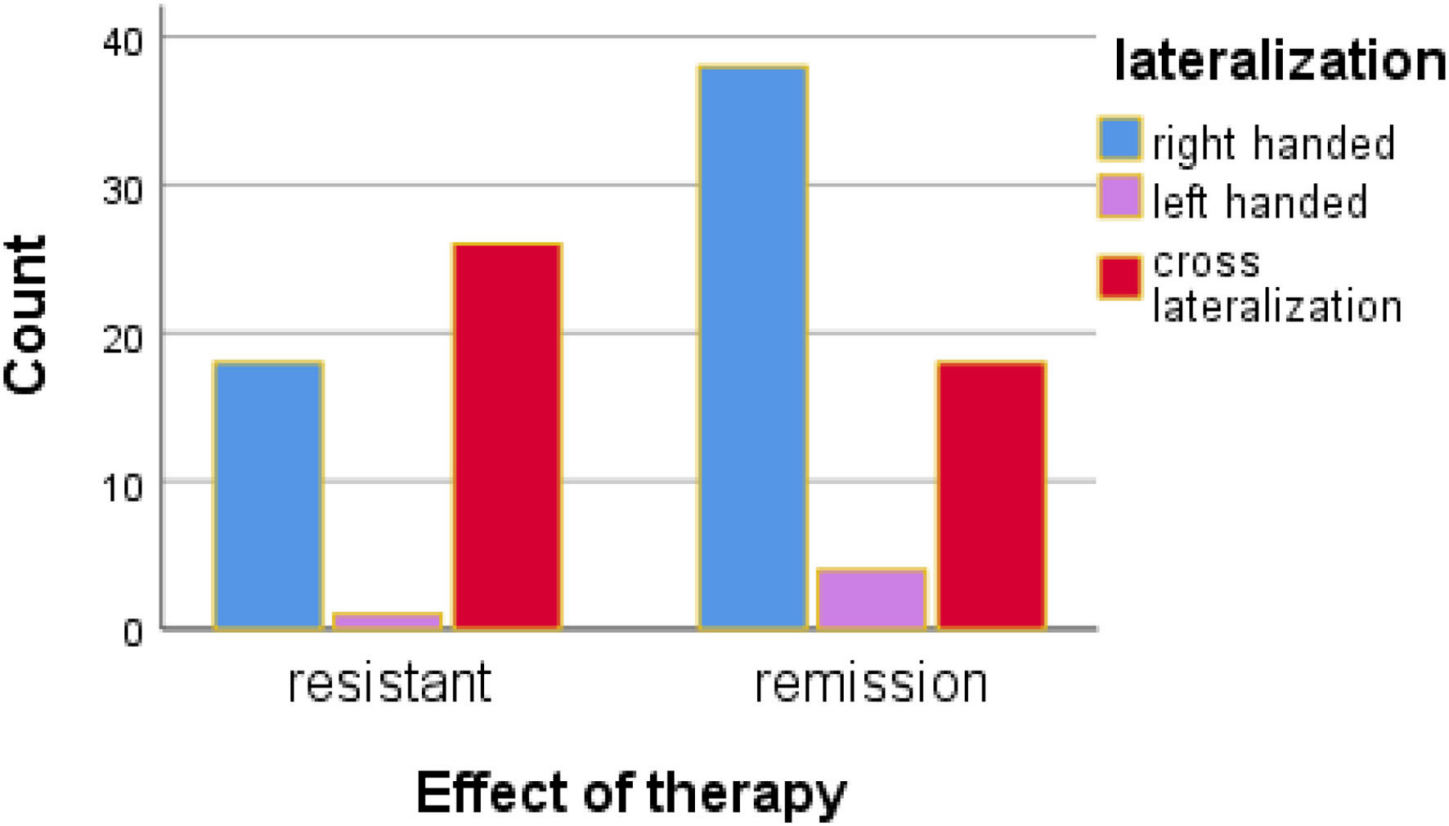
The relationship between functional lateralization /pure and cros-dominance/ and treatment effectiveness.

We find a statistically significant difference between the two groups of patients shown in Figure 1 and Table 5 (*p* < 0.05, ^*^).

**Table 5:** Statistical significance of the differences between patients in the two groups.

	**Value**	**df**	**Asymptotic significance**
			**(two sided)**
Pearson Chi-square	8.427	2	0.015
Likelihood ratio	8.543	2	0.014
Linear-by-linear association	8.045	1	0.005
No of valid cases	105		

### Gender-Related Differences in Functional Lateralization Showed the Following Results

In 66 female patients, 28 (42.4%) are cross-dominant, 35 (53%) are right-handed, and 3 (4.5%) are left-handed.

In 39 male patients, 16 (41.0%) have mixed dominance, 21 (53.8%) are right-handed, and 2 (5.1%) are left-handed.

Our results do not show significant gender differences in the lateralization of brain processes.

Gender-associated differences in terms of only the lateralization of the dominance of the eye showed that in in 26 (39.4%) females, there is a dominance of the left eye and in 40 (59.6%), there is dominance of the right eye. In males, it was found that 18 (46.2%) has dominance of the left eye, and 21 (53.8%) has dominance of the right eye.

We find a little higher percentage of patients with left eye dominance in males than in females.

The conclusions from the analysis of our results regarding the lateralization of brain processes in patients with schizophrenia show that there is an increased percentage of patients with cross-dominance in the general group of patients with schizophrenia. We registered an increased percentage of patients with a dominant left eye in the patients with schizophrenia we observed. We found a statistically significant difference in the dominance of the left eye with a pronounced predominance in patients with resistance to treatment. The increased percentage of patients with cross-dominance is due to patients with left-sided dominance of the eye.

We did not find an increased percentage of patients with a dominant left arm. We register a higher percentage of patients with dominance of the left leg compared with dominance of the left arm.

## Discussion

The use of a trimodal classification model to assess the lateralization of brain processes showed that our study did not reveal gender-related differences, unlike some studies that show one (40). We do not find gender-related differences in the lateralization of cerebral hemisphere dominance. Literature data show that there is a general prevalence of left-handed among males. A study conducted in Greece found that it was observed in 8.26% of men and 6.41% of females (40). In our sample, the difference between left-handed male and female left-handed patients is insignificant. This can be interpreted in the context of the observation of other authors that these gender differences are not universal (41).

Our results do not support the data of other authors on the predominance of left-handedness in patients with schizophrenia (17, 18). We find a high percentage of patients with mixed lateralization (i.e., cross use of hand, foot, or eye). Most likely, some studies did not look at cross-lateralization, but only left-handed or right-handed dominance, and did not use a trimodal classification, but a bimodal one. If we have to use the bimodal classification and look at the lateralization in terms of hand dominance, we found that seven of the patients have a dominant left hand. This makes 6.7% of all patients included in our study. The range of reported results of left-handedness in patients with schizophrenia varies from 7 to 31%. Our result is in the lower range of reported cases, and we do not confirm the data of other authors for high rates of dominance of the left hand in patients with schizophrenia—up to 40% (18). Other analyses show that differences in lateralization are not universal, and there are many divergent results (41). The results of other analyses indicate that left-handed people have a predominance in patients with schizophrenia. Using a bimodal classification, our study did not support these studies (17, 18). We find a low percentage of patients with a dominant left arm that is comparable with those in the general population. These results support the data of other researchers on the lack of differences in left-handed dominance in patients with schizophrenia and the general population (30). Our results show that when using a trimodal classification in patients with resistant symptoms, those with cross-dominance (mixed lateralization) predominate. We support the researchers' view that mixed dominance is typical of patients with schizophrenia, as we find that more than 40% of all patients have cross-dominance (28). The results of other studies also indicate that mixed or cross-dominance is three times more common in patients with schizophrenia than in the general population (30, 42). The data obtained by us (twice as many), in patients with mixed lateralization with resistance in comparison with those in remission, support the assessment of other authors for the more severe course of the disease in those with cross-dominance (20, 21, 30).

On the other hand, neurophysiological studies to assess the lateralization of background activity in the EEG also do not show the presence of dominant hemisphere in patients with schizophrenia with and without therapy, which also indirectly supports the idea of mixed dominance in patients with schizophrenia (43). Our study shows that the high frequency of cross-dominance we found is due to the left lateralization of eye dominance. Analyses by other authors also found left-sided dominance of the eye in patients with schizophrenia, which is more pronounced in male patients (32). Our study showed a slightly higher percentage of men with left eye dominance than women.

One of the limitations of our study is that due to the presence of psychotic production—delusions and hallucinations (we analyze patients with resistance), it was not possible to assess in detail the continuity of dominance with analysis of mixed forms and their weight in the assessment of refractoriness. On the other hand, an additional problem is the formation of social dysfunction with concomitant change in behavior in many patients, which also leads to a reduction in daily functioning and activity in the use of hands and eyes. A third important factor is the presence of additional comorbidity due to the fact that these patients do not conduct systematic treatment of their health problems, including the care of the visual analyzer, which would call into question attempts for accurate and detailed assessment.

Our study provides guidance for future studies related to resistance and disconnection syndrome in association with the fact that the very presence of cross-dominance raises the question of easier disruption of information flows in the crossover of the neural network associated with them.

## Conclusion

We found an association between the presence of cross-dominance and the treatment resistance in patients with schizophrenia. We reported that cross-dominance was found in more than half of the patients with resistant schizophrenia. This increased frequency of mixed dominance in them is due to left-sided dominance of the eye. These observations of ours can also be of practical use. The assessment of the dominant eye as well as a more in-depth analysis of the overall lateralization of dominance would provide guidance for early assessment of future development of resistance during treatment. Opportunities for early assessment provide a basis for seeking complex methods of treatment earlier in the course of the development of the schizophrenic process in order to prevent psychosocial complications associated with resistance.

## Data Availability Statement

The raw data supporting the conclusions of this article will be made available by the authors, without undue reservation.

## Ethics Statement

The studies involving human participants were reviewed and approved by Ethics Commission of the University Hospital for Active Treatment, Stara Zagora. The patients/participants provided their written informed consent to participate in this study.

## Author Contributions

The author confirms being the sole contributor of this work and has approved it for publication.

## Conflict of Interest

The author declares that the research was conducted in the absence of any commercial or financial relationships that could be construed as a potential conflict of interest.

## Publisher's Note

All claims expressed in this article are solely those of the authors and do not necessarily represent those of their affiliated organizations, or those of the publisher, the editors and the reviewers. Any product that may be evaluated in this article, or claim that may be made by its manufacturer, is not guaranteed or endorsed by the publisher.
